# A non-sense mutation in the putative anti-mutator gene *ada/alkA *of *Mycobacterium tuberculosis *and *M. bovis *isolates suggests convergent evolution

**DOI:** 10.1186/1471-2180-7-39

**Published:** 2007-05-16

**Authors:** Laurent X Nouvel, Tiago Dos Vultos, Eric Kassa-Kelembho, Jean Rauzier, Brigitte Gicquel

**Affiliations:** 1 Unité de Génétique Mycobactérienne, Institut Pasteur, 28 rue du Dr. Roux, 75724 Paris Cedex, France; 2 Laboratoire des Mycobactéries, Institut Pasteur, Bangui, Central African Republic

## Abstract

**Background:**

Previous studies have suggested that variations in DNA repair genes of W-Beijing strains may have led to transient mutator phenotypes which in turn may have contributed to host adaptation of this strain family. Single nucleotide polymorphism (SNP) in the DNA repair gene *mutT*1 was identified in MDR-prone strains from the Central African Republic. A *Mycobacteriumtuberculosis *H37Rv mutant inactivated in two DNA repair genes, namely *ada/alkA *and *ogt*, was shown to display a hypermutator phenotype. We then looked for polymorphisms in these genes in Central African Republic strains (CAR).

**Results:**

In this study, 55 MDR and 194 non-MDR strains were analyzed. Variations in DNA repair genes *ada/alkA *and *ogt *were identified. Among them, by comparison to *M. tuberculosis *published sequences, we found a non-sense variation in *ada/alkA *gene which was also observed in *M. bovis *AF2122 strain. SNPs that are present in the adjacent regions to the amber variation are different in *M. bovis *and in *M. tuberculosis *strain.

**Conclusion:**

An Amber codon was found in the *ada/alkA *locus of clustered *M. tuberculosis *isolates and in *M. bovis *strain AF2122. This is likely due to convergent evolution because SNP differences between strains are incompatible with horizontal transfer of an entire gene. This suggests that such a variation may confer a selective advantage and be implicated in hypermutator phenotype expression, which in turn contributes to adaptation to environmental changes.

## Background

With more than 2 million deaths a year, tuberculosis (TB) remains a major public health problem. As one-third of the world's population is infected, *Mycobacterium tuberculosis *is probably the most widespread human pathogen and the number of TB cases is growing at a rate of 2% per year [1]. This bacterium is remarkably well-adapted to its host, in which it can persist for years without inducing symptoms. Despite its worldwide dissemination and presence in diverse host populations, the genetic variability of *M. tuberculosis *appears to be very low with the exception of hot spot regions in the genome that are associated with mobile elements [2-5].

Previous studies on W-Beijing family strains have identified variations in putative DNA repair genes (*mutT2*, *mutT4 *and *ogt*). These variations are found only in the W-Beijing family; they can therefore be considered to be specific markers of this family and suggest that a mutator phenotype, at least transient, may have contributed to the host adaptation of this family of strains [6].

Durbach et al. [7] co-inactivated DNA repair genes, namely *ada*/*alkA *and *ogt*, in strain H37Rv and showed that the resulting mutant displayed a mutator phenotype under nitrosative stress.

While studying the genetic diversity of *M. tuberculosis *isolates in the Central African Republic (CAR), we described an MDR-prone family characterized by a T family spoligotype and a synonymous variation in the DNA repair gene *mutT*1 [8]. This prompted us to screen our set of well-defined isolates from CAR [8,9] by sequencing putative anti-mutator genes including *ada/alkA *to identify other variations that could be markers of relevant clinical strains.

Comparisons of CAR strain sequences with published "*M. tuberculosis *complex" genome sequences revealed an SNP common to these strains and *M. bovis *strain AF2122. This suggests either horizontal DNA transfer between *M. bovis *and *M. tuberculosis *CAR strains or convergent evolution. Medigue et al. and Denamur et al. [10,11] demonstrated horizontal transfer of DNA repair genes in *E. coli*. To determine whether there has been transfer between *M. bovis *and *M. tuberculosis *strains, we looked at polymorphism in the regions upstream and downstream from this common SNP by PCR sequencing. We provide evidence that this SNP may have occurred twice independently and might therefore be an example of convergent evolution.

## Results

### Variations in the *ada/alkA* and *ogt* genes in a series of MDR isolates

Variations in *ada/alkA *and *ogt *genes were assessed in fifty-five MDR strains collected between 1993 and 2001 in CAR. PCR products corresponding to *ada/alkA *and those corresponding to *ogt *were obtained for each MDR CAR strain in our collection. Direct sequencing of PCR products revealed four variations in DNA repair genes: one in *ogt *and three in *ada/alkA *(table 1).

The *ogt *variant is a C to G substitution causing the replacement of a Threonine by a Serine at position 15 in Ogt, noted A CC→A GC (Thr15→Ser). This variation is carried by three CAR isolates. It has been previously observed in other strains and considered to be characteristic of Haarlem strains [6,12].

Nine strains carry an ACT→AAT (Thr337→Asn) variation, and five of these strains also display a TGG→TAG (Trp79→AMBER) variation. This is the first report of a variant involving a nonsense codon in *M. tuberculosis*. This raised the question of whether such variations also occurred in non-MDR strains.

### Polymorphism of *ada/alkA* in a series of non-MDR strains (Table 1)

Using the same sequencing approach, we searched for the presence of the nonsense variation TGG→TAG (Trp79→AMBER) and other *ada/alkA *or *ogt *polymorphisms in a set of 194 well-defined non-MDR strains isolated in CAR between April 15^th ^and August 15^th ^1998 from the cohort studied by Espinal et al. [9]: 138 of these isolates were found to carry sequence variations (Table 1). Twenty-four of the strains carry the ACT→AAT (Thr337→Asn) variation, including three that also carry the TGG→TAG (Trp79→AMBER).

We searched *ada/alkA *sequences in available "*M. tuberculosis *complex" genome sequences and found the TGG→TAG (Trp79→AMBER) variation in *M. bovis *strain AF2122. The spoligotypes of the CAR isolates with the TGG→TAG (Trp79→AMBER) variation indicate that they are Haarlem *M. tuberculosis *strains. This suggests that this Amber mutation has occurred independently in at least two different species of the "*M. tuberculosis *complex" or that it has been acquired by horizontal DNA transfer. To test these possibilities, we studied the polymorphisms of chromosomal regions flanking the TGG→TAG (Trp79→AMBER) *ada/alkA *SNP.

We compared regions flanking the Amber variation in *M. tuberculosis *strains including (I) CAR isolates carrying the Thr337→Asn variation with or without the Amber variant, (II) H37Rv, (III) CDC1551 and (IV) W-Beijing 210 and in *M. bovis *strains including (V) RCA isolates, (VI) strain AF2122 and (VII) BCG Pasteur. In addition to the *ada/alkA *gene variations previously found in MDR CAR strains, we identified two further SNPs affecting the *ada/alkA *coding sequence: GCG→ACG (Ala11→Thr) only found in W-Beijing210 and CTG→CTA (Leu269→Leu) harboured by *M. bovis *AF2122 and BCG Pasteur. We also found six SNPs outside of the *ada/alkA *coding sequence: SNP1, SNP2 and SNP3 downstream from *ada/alkA*, and SNP4, SNP5 and SNP6 upstream from the gene (table 2). SNPs 2, 4, 5 and 6 were found in all strains except in H37Rv. SNP1 and the ATC→GTC (Ile12→Val) variation were found in all *M. bovis *strains but not in any CAR isolates of *M. tuberculosis*. The CTG→CTA (Leu269→Leu) variation is present only in *M. bovis *AF2122 and BCG Pasteur and SNP3 only in *M. bovis *AF2122. In contrast, CAR *M. tuberculosis *Amber variants also harbour the ACT→AAT (Thr337→Asn) variation and do not carry SNP1 or SNP3, or codon 12 and 269 modifications. Therefore, DNA polymorphisms identified in CAR *M. tuberculosis *Amber variant isolates differ from those observed in *M. bovis *strain AF2122 (Table 2). This is not compatible with the occurrence of the Amber variation in both groups being a consequence of horizontal transfer and allelic replacement of the entire *ada/alkA *gene. It is therefore more likely that independent events are responsible for the appearance of this variation in the two species, thus suggesting convergent evolution.

### Spoligotypes of *M. tuberculosis* strains and relatedness

**Figure 1:**
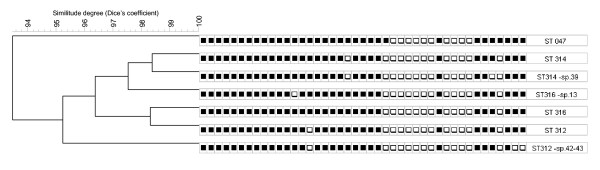
Dendrogram of the various spoligopatterns of strains harbouring *ada/alkA *variations at codon 337 or 337 and 79.

Spoligotyping of all these CAR isolates has been reported in Nouvel et al. [8]. Spoligotype clusters were named by their Shared Type number according to the spoligotype database SpolDB3 [13]. We considered the distribution of CAR Amber variant isolates among the clusters. They were found in three different clusters: (I) "ST047" (composed of 50 strains of which only one carries the Amber variation), "ST316 minus spacer 13" (five strains, all carrying the Amber variant, four of them being MDR) and "ST316" (22 strains, two carrying the Amber variation). The ACT→AAT (Thr337→Asn) variation alone (not associated with the Amber variant) is present in strains of clusters "ST047", "ST316", "ST312" and "ST312 minus spacers 42–43". These clusters share 93.47 to 98.4% spoligotype identity (Figure 1) and could reasonably be considered as a single family.

## Discussion

In our collection of CAR isolates, the *ada/alkA *gene variant ACT→AAT (Thr337→Asn) was found in 16 isolates, eight of which also carry the TGG→TAG (Trp79→AMBER) variation. This suggests that the ACT→AAT (Thr337→Asn) mutation appeared before the TGG→TAG (Trp79→AMBER) mutation.

**Figure 2:**
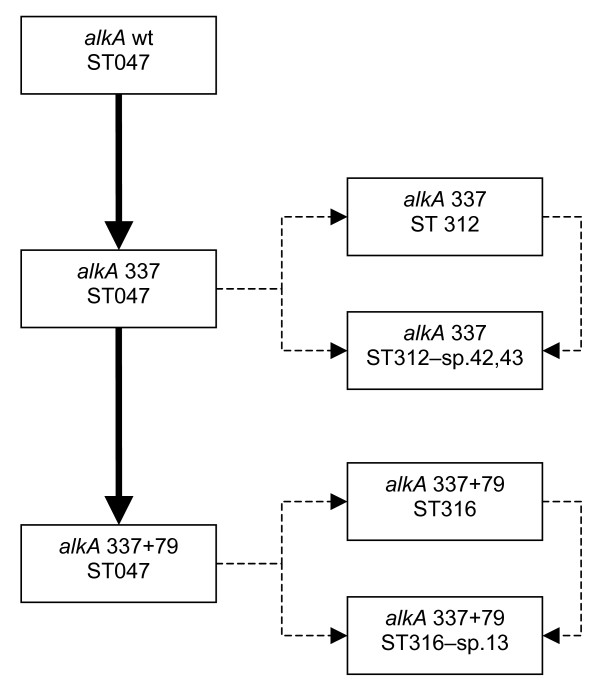
Probable emergence of *ada/alkA *variants.

Moreover, strains carrying only the ACT→AAT (Thr337→Asn) variation show spoligopatterns "ST047", "ST312", "ST314", "ST316", "ST312 without spacers 42–43", or "ST314 without spacers 39". Strains carrying both ACT→AAT (Thr337→Asn) and TGG→TAG (Trp79→AMBER) belong to "ST047", "ST316" or "ST316 without spacer 13". All these Shared Types are similar and differ by a small number of spacers. The spoligopatterns of these 16 strains classify them into the Haarlem family (similar to ST047). However, the strains with ACT→AAT (Thr337→Asn) variation with or without the TGG→TAG (Trp79→AMBER) variation do not carry variations in *ogt*: this suggests they are all derived from an ancient Haarlem family strain before the appearance of the *ogt *variants. We propose a genealogy of strains showing a Haarlem spoligotype and carrying the Amber variation in the *ada/alkA *gene (Figure 2). Further studies will be necessary to assess whether these strains are an emerging cluster in CAR.

The Amber variation in *ada/alka *is also found in *M. bovis *strain AF2122. This Amber variation is absent from the two *M. bovis *strains isolated in CAR analysed in this study (identified as *M. bovis *by biochemical tests and confirmed by spoligotyping). It is also absent from the *M. bovis *BCG Pasteur strain. Denamur *et al*. [11] demonstrated horizontal transfer of DNA repair genes between *E. coli *strains and showed that this led to the appearance of mutator phenotypes. Our SNP analysis of the *ada/alkA *DNA region excludes this possibility for the Amber variation. All *M. tuberculosis *strains carrying the Amber variant in our study population harbour the same SNPs; these SNPs are different from those found in *M. bovis*AF2122. This argues strongly against horizontal transfer of a region comprising the entire *ada/alka *gene. Therefore, the Amber variation probably occurred more than once independently and thus constitutes a case of convergent evolution. The acquisition of this variation may confer a selective advantage on the strain.

In several bacteria, the Ada and AlkA proteins are encoded by two distinct genes. The putative *ada/alkA *gene of *M. tuberculosis *is similar to the *ada *and *alkA *genes of *E. coli*, and is likely to be a fusion of the two genes [14]. Interestingly, the non-sense mutation we described in CAR isolates and in *M. bovis *AF2122 affects the Ada part of the gene. Presumably, the Amber mutation inactivates the *ada/alkA *fused gene and results in a mutator phenotype. Indeed, inactivation of this gene results in an increased mutation frequency in *M. tuberculosis *without any growth impairment *in vivo *[7]. Thus, the naturally occurring *ada/alkA *mutants we report here may have the advantage of a high mutation rate without any associated cost *in vivo*. Alternatively, the production of a truncated Ada protein may constitutively activate adaptive responses, as has been demonstrated in *E. coli *[15]. In particular, it may provide an improved response to stress *in vivo*, for instance in the phagosome of activated macrophages [16].

## Conclusion

An Amber stop-codon in *ada/alkA *was detected in some *M. tuberculosis *Haarlem isolates from CAR and also in *M. bovis *strain AF2122. This Amber variation appears not to have spread by horizontal gene transfer; this could be a case of convergent evolution between *M. tuberculosis *and *M. bovis*. This suggested that acquisition of the variation may confer a selective advantage. The *ada/alkA *genetic locus of *M. tuberculosis *has been implicated in mutator phenotype expression [7]. The variant we report may therefore contribute to adaptation to environmental changes. In addition, we describe polymorphisms in DNA repair genes in *M. tuberculosis *isolates and these could be used for strain characterization.

## Methods

### Strains analyzed

The collection of strains isolated in the Central African Republic was previously described in Nouvel et al. [8]. Briefly, it includes 55 MDR *Mycobacterium tuberculosis *strains from different patients and 194 non-MDR strains from the cohort studied by Espinal et al. [9] for which a subculture was obtained (192 *M. tuberculosis *and 2 *M. bovis *strains). Spoligotyping, a method for typing strains of *M. tuberculosis *complex, was performed as previously described [2,17]. Spoligopatterns are identified by Shared Type number (ST) according to spolDB3 nomenclature [13].

### Sequences of *ada/alkA* and *ogt*

Primers were designed to amplify putative *mut *genes: *ogt *(5'-CAGCGCTCGCTGGCGCC -3', 5'-GACTCAGCCGCTCGCGA-3'), and *ada/alkA *(5'-AGCCGCGTAGGTAACCT-3', 5'-TGCTCGAGCATCCGCAG-3') and (5'-CGCATGCAGACCGCCCG-3', 5'-CACTGCACGTTGCCGAC-3').

DNA sequences of the amplified fragments were determined directly by using the dideoxy chain-termination method with the Big Dye Terminator Cycle Sequencing Kit (Perkin Elmer Applied Biosystems, Courtaboeuf, France) on a GeneAmp polymerase chain reaction (PCR) system9600 (Perkin Elmer) and run on a DNA analysis system model3100 (Applied Biosystems).

Sequences of genes from *M. tuberculosis *H37Rv, MT210 and CDC1551, and *M. bovis *AF2122 were obtained from published sequences [14,18,19] available on the TIGR web site [20].

### Computer-assisted analysis of dendrograms

Autoradiographs were scanned, and the resulting spoligotype data for each mycobacterial isolate was analysed using the Windows version of the computer-assisted BioNumerics software (Applied Maths, Kortrijik, Belgium). Each positive spot was defined as a band. The software then clustered strains with the same spoligotyping patterns. Dendrograms were constructed for the spoligotypes according to degree of similitude (Dice's coefficient) and comparison with known spoligotypes [13,21,22].

### Comparison of regions flanking *ada/alkA*

A 20 kb DNA region comprising the *ada/alkA *coding sequence in *M. tuberculosis *H37Rv was aligned with the corresponding regions in *M. bovis *AF2122 [19], *M. tuberculosis *CDC1551, and *M. tuberculosis *MT210 to identify variations: 9 SNPs were found. Primers were designed to correspond to these sequences (as described above). *M. tuberculosis *H37Rv, *M. bovis *BCG Pasteur and 16 strains from Bangui (including 2 *M. bovis *strains according to biochemical and spoligotyping markers) were sequenced for these SNPs. A DNA parsimony analysis was used to generate a phylogenetic tree.

## Abbreviations

CAR : Central African Republic

*M*. : *Mycobacterium*

MDR : Multidrug resistant

SNP : Single Nucleotide Polymorphism

ST : Shared Type

TB : tuberculosis

## Authors' contributions

LXN carried out the molecular marker study, analyzed results, participated in DNA sequencing, sequence alignment work, and drafted the manuscript. TDV participated in the analysis of results and helped to draft the manuscript. EKK provided genomic DNA of CAR isolates, conducted epidemiological studies and contributed to the analysis of results. JR carried out DNA sequencing and participated in the analysis of results. BG conceived the study, participated in its design and coordination and helped draft the manuscript. All authors have read and approved the final manuscript.

## Figures and Tables

**Table 1:** *ada/alkA *variants

	***ogt***	***ada/alkA***				
		
**N° of strains**	***Codon 15***	***Codon 11***	***Codon 12***	***Codon 79***	***Codon 254***	***Codon 337***
		**among MDR**				

41	wt	wt	Wt	wt	wt	wt
3	ACC→AGC (Thr 15→Ser)					
2	wt	wt	ATC→GTC (Ile 12→Val)	wt	wt	wt
4	wt	wt	Wt	wt	wt	ACT→AAT (Thr 337→Asn)
5	wt	wt	Wt	TGG→TAG (Trp 79→AMBER)	wt	ACT→AAT (Thr 337→Asn)
		**among non-MDR**				

56	wt	wt	Wt	wt	wt	wt
105	ACC→AGC (Thr 15→Ser)	wt	Wt	wt	wt	wt
1	wt	wt	ATC→GTC (Ile 12→Val)	wt	wt	wt
7	wt	wt	ATC→GTC (Ile 12→Val)	wt	wt	wt
1	wt	wt	ATC→GTC (Ile 12→Val)	wt	Pro CCC 254 Pro CCA	wt
21	wt	wt	Wt	wt	wt	ACT→AAT (Thr 337→Asn)
3	wt	wt	Wt	TGG→TAG (Trp 79→AMBER)	wt	ACT→AAT (Thr 337→Asn)

wt: wild type (H37Rv)

**Table 2:** SNPs in the *alkA *region

	***Downstream region***			← ***ada/alkA***^b^					***Upstream region***
					
**SNP position**^a^	***SNP1 *1471.218**	***SNP2 *1471.659**	***SNP3 *1477.028**	***Codon 337 *1478.108**	***Codon 269 *1478.312**	***Codon 79 *1478.883**	***Codon 12 *1479.085**	***Codon 11 *1479.088**	***SNPs 4, 5, 6 *1480.972, 1482.627, 1484.708**
**Strains**	**In CAR strains**								

***M.tb alkA *79+337**^c^	wt	C→T	wt	ACT→AAT	wt	TGG→TAG	wt	wt	T→C, T→C, A→C
***M.tb alkA *337**^d^	wt	C→T	wt	ACT→AAT	wt	wt	wt	wt	T→C, T→C, A→C
***M.bv *CAR**^e^	C→T	C→T	wt	wt	wt	wt	ATC→GTC	wt	T→C, T→C, A→C
	**in other strains**								

***M.bv *AF2122**	C→T	C→T	C→-	wt	CTG→CTA	TGG→TAG	ATC→GTC	wt	T→C, T→C, A→C
***M. bv *BCG**	C→T	C→T	wt	wt	CTG→CTA	wt	ATC→GTC	wt	T→C, T→C, A→C
***M.tb *210**	wt	C→T	wt	wt	wt	wt	ATC→GTC	GCG→ACG	T→C, T→C, A→C
***M.tb *CDC1551**	wt	C→T	wt	wt	wt	wt	wt	wt	T→C, T→C, A→C
***M.tb *H37Rv**	wt	wt	wt	wt	wt	wt	wt	wt	wt

^a ^SNP position = base position according to the numbering of the H37Rv genome [18]

^b ^*ada/alkA *coding sequence is on the minus strand

^c ^isolates from CAR that harbour variations at codon 79 and 337 in *ada/alkA*, 8/8 isolates sequenced

^d ^isolates from CAR that harbour variations at codon 337 in *ada/alkA*, 5/25 isolates sequenced

^e ^*M. bovis *isolate from CAR sequenced (one isolate)

CAR, Central African Republic ; *M.bv*, *Mycobacterium bovis *; *M.tb*, *Mycobacterium tuberculosis *; wt, wild type (H37Rv).
